# Supplementary material for: Comparative evaluation of sensititre YeastOne and VITEK2 antifungal susceptibility tests with CLSI broth microdilution method of clinical Cryptococcus isolates in Taiwan
Source: Microbiol Spectr. 2024 Dec 19;13(2):e02117-24. doi: 10.1128/spectrum.02117-24 (PMC11792495; doi:10.1128/spectrum.02117-24)
Supplement: Supplemental material — Tables S1 to S3. [file spectrum.02117-24-s0001.pdf]

Supplementary Table 1 Epidemiological cutoff values for *in vitro* susceptibility testing of *Cryptococcus* species

Species	Drug	ECV by CLSI (mg/L)	Clinical breakpoint by EUCAST (mg/L)	ECV by EUCAST (mg/L)
C. neoformans				
	AMB	0.5	1	1
	5FC	8		
	FLC	8		
	VRC	0.25		0.5
	ITC	0.25		
	PSC	0.25		0.5
C. gattii				
	AMB	0.5		0.5
	5FC	4		
	FLC	16		
	VRC	0.5		
	ITC	0.5		
	PSC			1

Abbreviations: ECV, Epidemiological cutoff value; CLSI, Clinical and Laboratory Standards Institute; EUCAST, European Committee on Antimicrobial Susceptibility Testing; AMB, amphotericin B; 5FC, flucytosine; FLC, fluconazole; VRC, voriconazole; ITC, itraconazole; PSC, posaconazole.

Supplementary Table 2 ***In vitro*** **antifungal susceptibilities of 44** ***Cryptococcus neoformans*** **of different sequence types determined by CLSI broth microdilution method.**

**Drug**	**MIC (µg/*ml*)**										**Non-WT**	**Non-WT**
**Sequence type**	**< 0.03**	**0.03**	**0.06**	**0.12**	**0.25**	**0.5**	**1**	**2**	**4**	**8**	**CLSI (%)**	**EUCAST (%)**
AMB												
ST 5						30	8				8 (21)	0
Non-ST 5						3	3				3 (50)	0
5FC												
ST 5								1	18	19	0	
Non-ST 5								1	2	3	0	
FLC												
ST 5						1	1	5	25	6	0	
Non-ST 5								3	3		0	
VRC												
ST 5	2	11	21	4							0	0
Non-ST 5		2	4								0	0
ITC												
ST 5	10	6	15	7							0	0
Non-ST 5	2	3	1								0	0
PSC												
ST 5		1	1	26	10						0	0
Non-ST 5			2	4							0	0

ISC											
ST 5	2		10	18	6	2					
Non-ST 5			3	3							
MGX											
ST 5					1	1		8	21	6	1
Non-ST 5					1				3	2	

Abbreviations: CLSI, Clinical and Laboratory Standards Institute; WT, wild type; EUCAST, European Committee on Antimicrobial Susceptibility Testing; ST, sequence type; AMB, amphotericin B; 5FC, flucytosine; FLC, fluconazole; VRC, voriconazole; ITC, itraconazole; PSC, posaconazole; ISC, isavuconazole; MGX, manogepix.

Supplementary Table 3 ***In vitro*** **antifungal susceptibilities of 44** ***Cryptococcus neoformans*** **isolates with and without ERG11 mutation determined by CLSI broth microdilution method**

**Drug**	**MIC (μg/*ml*)**										**Non-WT**	**Non-WT**
**ERG11 mutation**	**< 0.03**	**0.03**	**0.06**	**0.12**	**0.25**	**0.5**	**1**	**2**	**4**	**8**	**CLSI (%)**	**EUCAST (%)**
AMB												
ERG11 mutation						31	8				8 (18)	0
No mutation						2	3				3 (60)	0
5FC												
ERG11 mutation								1	18	20	0	
No mutation								1	2	2	0	
FLC												
ERG11 mutation						1	1	5	26	6	0	
No mutation								3	2		0	
VRC												
ERG11 mutation	2	12	21	4							0	0
No mutation		1	4								0	0
ITC												
ERG11 mutation	10	7	15	7							0	0
No mutation	2	2	1								0	0
PSC												
ERG11 mutation		1	1	27	10						0	0
No mutation			2	3							0	0

ISC											
ERG11 mutation	2		10	19	6	2					
No mutation			3	2							
MGX											
ERG11 mutation					1	1		8	22	6	1
No mutation					1				2	2	

Abbreviations: CLSI, Clinical and Laboratory Standards Institute; WT, wild type; EUCAST, European Committee on Antimicrobial Susceptibility Testing; AMB, amphotericin B; 5FC, flucytosine; FLC, fluconazole; VRC, voriconazole; ITC, itraconazole; PSC, posaconazole; ISC, isavuconazole; MGX, manogepix.
